# COVID-19 Prevalence among Healthcare Workers. A Systematic Review and Meta-Analysis

**DOI:** 10.3390/ijerph19010146

**Published:** 2021-12-23

**Authors:** Tafadzwa Dzinamarira, Grant Murewanhema, Malizgani Mhango, Patrick Gad Iradukunda, Itai Chitungo, Moreblessing Mashora, Pelagia Makanda, James Atwine, Munashe Chimene, Elliot Mbunge, Munyaradzi Paul Mapingure, Innocent Chingombe, Godfrey Musuka, Sphamandla Josias Nkambule, Bernard Ngara

**Affiliations:** 1 School of Health Systems & Public Health, University of Pretoria, Pretoria 0002, South Africa; 2 ICAP at Columbia University, Harare, Zimbabwe; mpm2189@cumc.columbia.edu (M.P.M.); ic2421@cumc.columbia.edu (I.C.); gm2660@cumc.columbia.edu (G.M.); 3 Unit of Obstetrics and Gynaecology, Department of Primary Health Care Sciences, Faculty of Medicine and Health Sciences, University of Zimbabwe, Harare, Zimbabwe; gmurewanhema@yahoo.com; 4 School of Public Health, University of Western Cape, Cape Town 7535, South Africa; 4012753@myuwc.ac.za; 5 London School of Hygiene and Tropical Medicine, University of London, London WC1E 7HT, UK; gadpatrickiradukunda@gmail.com; 6 Faculty of Medicine, College of Medicine and Health Sciences, University of Zimbabwe, Harare, Zimbabwe; ichitungo@medsch.uz.ac.zw (I.C.); bernardngara4@gmail.com (B.N.); 7 Department of Public Health, Mount Kenya University, Kigali 00100, Rwanda; mcmashora@gmail.com; 8 Department of Medicine, Jinzhou Medical University, Jinzhou 121001, China; makandapelagia@gmail.com (P.M.); jamesatwine90@gmail.com (J.A.); 9 Department of Health Sciences, Africa University, Mutare, Zimbabwe; munashe24@gmail.com; 10 Department of Information Technology, Faculty of Accounting and Informatics, Durban University of Technology, Durban 4000, South Africa; mbungeelliot@gmail.com; 11 Department of Public Health Medicine, School of Nursing and Public Health, University of KwaZulu-Natal, Durban 4001, South Africa; 210501689@stu.ukzn.ac.za

**Keywords:** healthcare worker, COVID-19, systematic review, meta-analysis, SARS-CoV-2

## Abstract

Understanding the burden of SARS-CoV-2 infections among healthcare workers is a critical component to inform occupational health policy and strategy. We conducted a systematic review and meta-analysis to map and analayse the available global evidence on the prevalence of SARS-CoV-2 infections among healthcare workers. The random-effects adjusted pooled prevalence of COVID-19 among those studies that conducted the test using the antibody (Ab) method was 7% [95% CI: 3 to 17%]. The random-effects adjusted pooled prevalence of COVID-19 among those studies that conducted the test using the PCR method was 11% [95% CI: 7 to 16%]. We found the burden of COVID-19 among healthcare workers to be quite significant and therefore a cause for global health concern. Furthermore, COVID-19 infections among healthcare workers affect service delivery through workers’ sick leave, the isolation of confirmed cases and quarantine of contacts, all of which place significant strain on an already shrunken health workforce.

## 1. Introduction

The COVID-19 pandemic, which the world has been battling with for over one and a half years now, brought unprecedented challenges for healthcare systems [1,2]. Of particular concern is the aggravated risk of infection to frontline healthcare workers (HCWs) compared to other infectious diseases [3]. This places additional requirements of personal protective equipment (PPE) and infection prevention and control (IPC) measures on healthcare systems, some of which are already strained and struggling with healthcare settings [4].

HCW COVID-19 infections lead to shortages of HCWs due to isolation and treatment periods, quarantining of contacts, hospitalization, mortality and the prolonged period from COVID-19. Countries such as the United Kingdom, USA, France, Italy and South Africa reported significant numbers of HCW infections and deaths during the early waves of the pandemic, resulting in a significant strain on human resources [5,6]. As powerful countries lose their HCWs due to death, morbidity or attrition from fear of contracting COVID-19, they attract HCWs from low-to-middle income countries (LMICs). LMICs offer less competitive working conditions and remuneration packages, and as countries lose their HCWs, an even bigger deficiency of HCWs will arise in these countries, which have been experiencing significant brain drain over the years, and struggling with human resources shortages [7]. Earlier COVID-19 deaths, which mainly occurred in older age groups above 50 years of age, sometimes resulted in the loss of significantly experienced HCWs, who act as pillars for teaching and providing mentorship to younger HCWs.

Similar to the general population, risk factors for developing symptomatic COVID-19 HCWs, the risk of severe disease and hospitalization, intensive care unit admission and death differ according to individual and population level characteristics. Based on findings from previous studies, elderly patients, those with chronic comorbidities such as diabetes mellitus and hypertension, the obese and those with other vulnerabilities are at a much higher risk of adverse outcomes from COVID-19 compared to the general population [8,9,10]. These patients are at an additional risk of thrombotic events within the first 3–6 months of COVID-19 diagnosis, especially if they are admitted into intensive care units or suffer from cancers [11]. Particular attention needs to be paid to their D-dimers, lactate dehydrogenase, white blood cell counts and lymphocyte counts [11]. These groups are key targets for intervention to reduce incident infections and mortality, such as COVID-19 vaccinations, which have become the crux of prevention, as well as standard IPC measures. Understanding the burden of SARS-CoV-2 infections among HCWs is a critical component to inform occupational health policy and strategy, including work placements stratified by differential risk, phased vaccination programs where supplies are inadequate, the distribution of healthcare resources such as PPE, and insurance policies. Over a year and a half into the pandemic, several studies have been published in these areas, but tend to focus on particular geographic areas. To widen the evidence base and enhance the understanding of the burden of COVID-19 among HCWs, we carried out a systematic review and meta-analysis. Our key objectives were to map and analyse the available evidence on the burden of SARS-CoV-2 infections among HCWs.

## 2. Methodology

PROSPERO registration number: CRD42020193508 available for public comments via the link below https://www.crd.york.ac.uk/prospero/display_record.php?ID=CRD42020193508) (accessed on 7 May 2021).

### 2.1. Study Design

This is a sub-study of a systematic review on COVID-19 risk factors among HCWs; the protocol was developed and published *a priori* [12]. The methodology was conducted and reported in accordance with the reporting guideline provided in the Preferred Reporting Items for Systematic Reviews and Meta-Analysis Protocols (PRISMA-P) statement [13].

### 2.2. Search Strategy, Study Selection and Data Extraction

Systematic literature searches were performed using the EBSCOhost platform, by searching the following terms through the Academic Health source: nursing/academic edition, CINAHL with full text, Embase, PubMed, MEDLINE, Science Direct databases, and Google Scholar. In addition, we also searched the China National Knowledge Infrastructure (CKNI) and the World Health Organization (WHO) library databases for relevant studies. Searches were conducted between 19 July 2021–10 August 2021. The search strategy published in the protocol [12] was adopted and was used as is. MM and IC conducted title, abstract and full text screening for EBSCo Host while PG and JA screened articles from CKNI. The study eligibility criteria are available in the published protocol [12]. Two reviewers working independently (MM and IC) and in duplicate extracted essential data into the standardised tables, and a third reviewer (TD) verified the data. All studies were managed in EndNote 20 at abstract and full text screening stages. From the included studies, the reported SARS-CoV-2 data per country were captured and used to map the distribution of healthcare workers affected with SARS-CoV-2 using QGIS 3.12.

### 2.3. Assessment of Study Quality and Risk of Bias

We employed the GRADE approach to obtain the rate the quality of the body of evidence for included studies. While there is no formal guidance for GRADE in systematic reviews of prevalence, we followed guidance from Migliavaca et al. [14] and employed the GRADE for baseline risk or overall prognosis tool [15]. Three investigators (MM, IC and MCM) assessed the included studies’ quality independently. Any disagreements were settled through a discussion among the investigators. Meta-biases were assessed using funnel plots to detect potential reporting biases and small-study effects [16] and complemented with the Egger regression test [17].

### 2.4. Data Synthesis and Statistical Analysis

A bilateral significance level of less than 0.05 was considered to be statistically significant. All analyses were performed using Meta and Metasens statistical packages available in R version 4.2.1 software package. The command functions described by Balduzzi et al. were employed [18].

## 3. Results

Our initial search resulted in 2448 articles overall. After removing 46 duplicates and adding 12 articles using a hand search, 2414 articles proceeded to the title screening phase. Among these, 2021 articles were excluded, and 393 articles proceeded to abstract review. Among these, 265 were excluded (Supplementary File S1). A total of 128 full text articles (Supplementary File S2) were screened for eligibility, 81 were excluded and 47 eligible studies were ultimately included in this meta-analysis. The included studies were conducted in America, Europe and Asia. Of the studies included, 22% were of the cohort study design and 25% were of cross-sectional study design. The distribution of healthcare workers affected with SARS-CoV-2 in the different countries included in this review is shown in Supplementary File S3. The distribution map shows that the United States of America, Mexico, China, Denmark and Italy are among the countries that reported the highest SARS-CoV-2 infections among healthcare workers. In contrast, Austria, Egypt and Canada reported the lowest SARS-CoV-2 infections among healthcare workers as indicated with light HCW-legend in the distribution map (Supplementary File S3). More details on the included studies characteristics are available in Supplementary File S4. Of the studies, 14 were graded as high quality, 21 as moderate quality, and 12 as low quality. More details are presented in Supplementary File S4.

**Figure 1:**
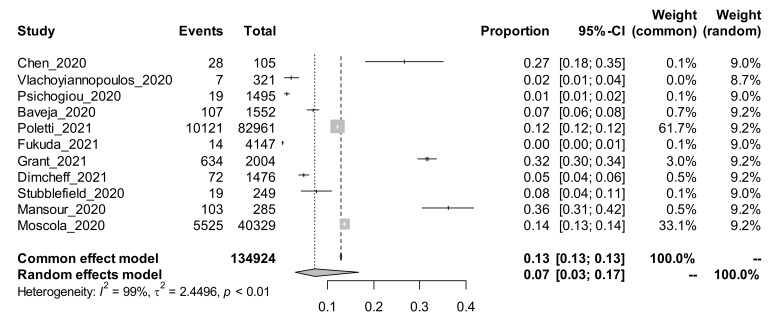
Forest plot showing the prevalence of COVID-19 among studies using the antibody method of test.

**Figure 2:**
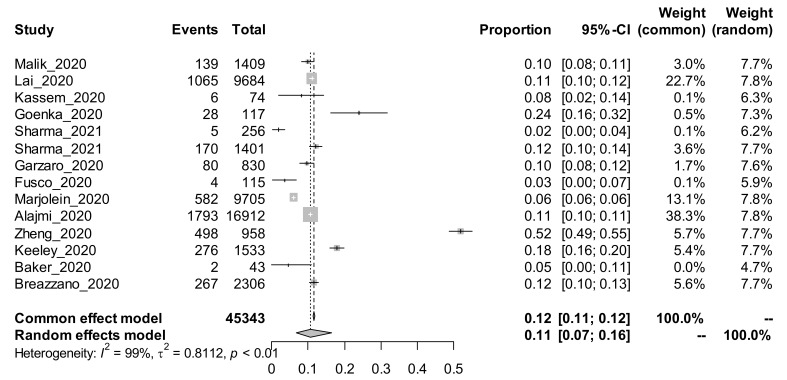
Forest plot showing the prevalence of COVID-19 among studies using the PCR method of test.

Among the 25 studies using the cross-sectional study design and estimating the prevalence of COVID-19, a subgroup analysis was performed based on the method of COVID-19 tests. The random-effects adjusted pooled prevalence of COVID-19 among those studies that conducted the test using the antibody (Ab) method was 7% [95% CI: 3 to 17%]. The random-effects adjusted pooled prevalence of COVID-19 among those studies that conducted the test using the PCR method was 11% [95% CI: 7 to 16%]. The forest plots for the subgroup analysis using the COVID-19 test method are shown in Figure 1 and Figure 2.

**Figure 3:**
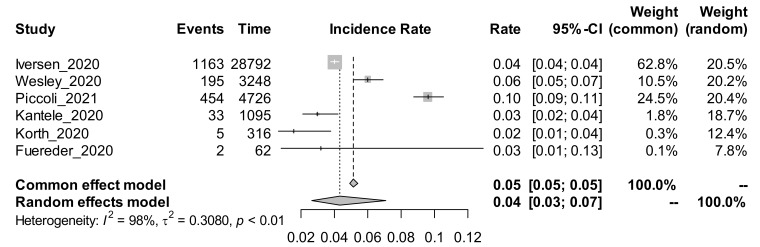
Forest plot showing the incidence rate of COVID-19 among studies using the antibody method of test.

**Figure 4:**
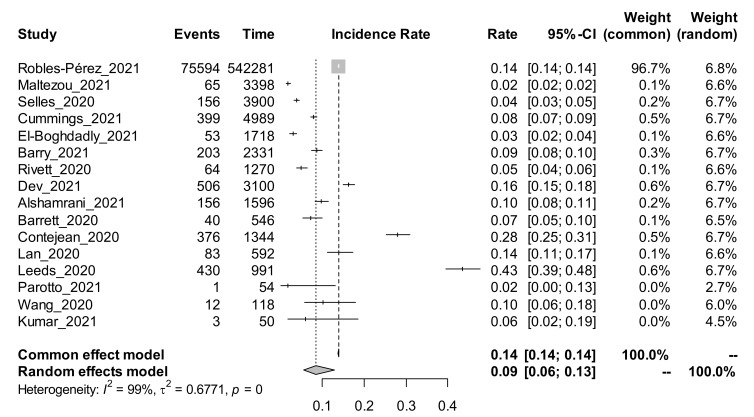
Forest plot showing the incidence rate of COVID-19 among studies using the PCR method of test.

Among the 22 studies using the cohort study design, and estimating the incidence rate of COVID-19, a subgroup analysis was performed based on the method of COVID-19 tests. The random-effects adjusted pooled incidence rate of COVID-19 among those studies that conducted the test using the antibody (Ab) method was 4% [95% CI: 3 to 7%]. The random effects adjusted pooled incidence rate of COVID-19 among those studies that conducted the test using the PCR method was 9% [95% CI: 6 to 13%]. The forest plots for the subgroup analysis by method of COVID-19 test are shown in Figure 3 and Figure 4.

**Figure 5:**
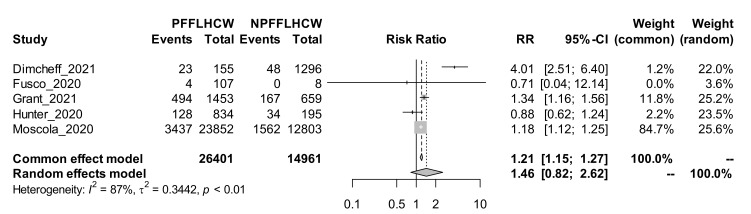
Forest plot showing the risk of COVID-19 between patient and non-patient facing HCWs.

A total of 5 studies that determine incidence rates by patient-facing or non-facing HCWs were included in the meta-analysis to determine the pooled difference in the incidence of COVID-19 among these subgroups of HCWs. Based on the fixed effect pooled estimate of the risk ratio, patient-facing was statistically significantly associated with incidence of COVID-19 at a 5% level. Patient-facing HCWs were 21% [95% CI: 15% to 27%], who were more likely to test positive for COVID-19 compared to non-patient-facing HCWs. Based on the random-effect pooled estimate of the risk ratio, patient facing was not statistically significantly associated with incidence of COVID-19 at 5% level. Patient facing HCW were 46% [95% CI: −18% to 16%] more likely to test positive for COVID-19 compared to non-patient facing HCWs. The forest plot showing the effect of facing patients are shown in Figure 5.

An egger’s test (*p* = 0.0001) and the asymmetric shape of the funnel plot implied a potential publication bias. More details are presented in Supplementary File S5. Funnel plots for assessing and accounting for small-study effects in the meta-analysis of: (1). prevalence of COVID-19 among studies using the Ab method of test (left-top panel); (2). prevalence of COVID-19 among studies using the PCR method of test (right-top panel); (3). incidence rate of COVID-19 among studies using the Ab method of test (left-bottom panel); (4). incidence rate of COVID-19 among studies using the PCR method of test; (right-bottom panel) are presented in Supplementary File S6.

## 4. Discussion

In this systematic review and meta-analysis, we estimated the burden of COVID-19 among HCWs to be quite significant, and therefore a cause for global health concern. Furthermore, COVID-19 infections among HCWs affect service delivery through workers’ sick leave, isolation of confirmed cases and quarantine of contacts, all of which place significant strain on an already shrunken health workforce. Apart from their impact on service delivery, COVID-19 can also have psychological impacts including fear, stress, depression and anxiety on HCWs, all of which can result in reduced work performance. A recently published survey noted moderate levels of emotional exhaustion and low levels of depersonalization among junior and middle-level doctors as significant occurrences during the COVID-19 pandemic [19].

In cross-sectional studies, the pooled prevalence of COVID-19 among HCWs tested using antibody tests was 7% (95% CI 3–17%), whilst it was 11% (95% CI 7–16%) among studies using PCR tests. Antibody tests were found, in a meta-analysis, to have poor sensitivity and specificity, and there is a lack of uniformity in their performance, with limited quality control, hence they no longer have a role in clinical practice [20]. The CDC advises against the use of antibody tests to determine the need for SARS-CoV-2 vaccination [21]. However, the WHO advises that they can provide useful information regarding the population’s exposure to SARS-CoV-2. Some communities may have much higher seroprevalence using antibody tests, but a significant proportion of the positive participants would not have reported any symptoms of COVID-19 in the preceding six months.

As an example, in a seroprevalence study in the general population in Zimbabwe, the seroprevalence was 19% (95% CI 15.1–23.5%) while the same was 53.0% (95% CI 49.6–56.4) in 2021; however, more than 50% of the patients had not reported any symptoms in the preceding six months [22]. In a meta-analysis of seroprevalence studies carried out in South America, a pooled estimate of 33.6% (95% CI 28.6–38.5%) was obtained; however, there were marked variations between studies, with seroprevalences as low as 70% in some settings [23]. In general, there is a marked heterogeneity in seroprevalence among studies conducted in the general population globally. It is also not clear how long this natural immunity conferred by these antibodies lasts, and their implications for global vaccination programmes. Given this gap, it remains important to go ahead with vaccination, especially of at-risk groups such as HCWs. Over time, the seroprevalence of COVID-19 diagnoses is expected to rise as greater proportions of the population become exposed to SARS-CoV-2; however, this will not necessarily transform into clinical significance. However, it can be assumed that at the time of this meta-analysis, significant proportions of both HCWs and the general population were still susceptible to COVID-19 as they lacked antibodies to signify previous exposure to SARS-CoV-2 infection.

PCR is the gold standard for diagnosing SARS-CoV-2 infection, and the high pooled prevalence of 11% noted here reflects higher infection rates among HCWs. However, PCR testing is expensive and not routinely afforded by low-to-middle income countries such as those in sub-Saharan Africa. Interestingly, our search did not retrieve any study from sub–Saharan Africa (SSA), where challenges of personal protective equipment (PPE) for HCWs are common, and therefore HCW COVID-19 infections might be more prevalent. The paucity of studies from this key region limits the generalizability of the findings; nevertheless, it still provides important information regarding the problem. The incidence rate of COVID-19 among HCWs was significantly high in the cohort studies using PCR testing at 9% (95% CI 6–13), compared to antibody testing, 4% 95% CI 3–7%). What is not established is whether these new infections among HCWs were contracted in the workplace or in the community, which would be important for formulating control strategies. If new infections are acquired in the community, then the HCWs should abide by the same infection prevention and control strategies adopted in the general population, including vaccination, physical distancing and wearing of face masks. However, if these new infections are acquired in the workplace, it speaks to the need to upscale preventive measures in the workplace, especially the provision of safe and adequate PPE, which would protect both the HCWs and their clients.

Five studies providing granular data for patient-facing and non-patient-facing HCWs were retrieved and a subgroup analysis was conducted. In this subgroup analysis, patient-facing HCWs were 21% more likely to test positive for COVID-19 compared to non-patient-facing HCWs. However, there was a marked heterogeneity in the studies, with an I2 of 87%, and in a random effects model, the difference was statistically insignificant with a wide 95% CI (46%, 95% CI −18–16%). Hence, there is still a need for higher quality studies with markedly reduced heterogeneity and adequate ability to determine whether patient-facing HCWs are at a significantly much higher risk of contracting COVID-19 than their non-patient facing contacts and determine the need for extra protection for this group. Given the biological plausibility of patient-facing HCWs being exposed to significantly higher loads of SARS-CoV-2 in clinical areas, conclusive evidence of a higher incidence rate is needed. Nevertheless, based on the findings of our meta-analyses, HCWs across the board must, to a greater extent, observe the universal precautions and infection prevention and control, and must continue to be prioritised for vaccination.

## 5. Conclusions

A level of heterogeneity is notable across all the cross-sectional and cohort studies involved in the different meta-analyses. This speaks to differences in sample sizes, methodologies and geographical variations. Therefore, we only discuss results from random effects meta-analysis since the heterogeneity cannot be explained as it is due to chance. Furthermore, we did not exclude studies based on low sample sizes. When prevalence is unknown, the standard sample size is 384 for statistical significance, although we included studies with smaller sample sizes. However, the results are still informative, providing a baseline risk of COVID-19 burden among HCWs and a baseline for future research. More uniform and high-powered studies are needed to address the question of whether patient-facing HCWs are at significantly higher risk than their non-patient facing counterparts, and whether there is a need for differentiated measures of protection between the groups, and the need for extra layers of protection for the patient-facing group. The other previously highlighted limitation is the lack of literature from SSA, which places significant limitations on the generalizability of the results from these meta-analyses to the continent. The other limitation is that these studies do not provide us with information on risk factors and outcomes, both of which are important for designing interventions to protect HCWs, including insurance policies, placements, and the provision of PPE. The risk factors will be discussed in a subsequent systematic review and meta-analysis. Due to the lack of uniformity and quality control, studies utilizing antibody tests lead to questions regarding reliability, validity and reproducibility. Hence, the 95% CIs for studies utilizing these tests were much higher compared to those studies utilizing PCR testing.

## Data Availability

All data related to this study are presented in the manuscript and supplementary files.

## Supplementary Materials

The following are available online at https://www.mdpi.com/article/10.3390/ijerph19010146/s1, Supplementary File S1: PRISMA Flow Diagram, Supplementary File S2: List of full text articles reviewed, Supplementary File S3: Distribution of COVID-19 burden among health care workers in included studies, Supplementary File S4: Characteristics of included studies, Supplementary File S5: Egger’s plots for assessing the presence of publication bias for the meta-analysis, Supplementary File S6: Presentation of findings for assessing and accounting for small-study effects.
