# Supplementary material for: COVID-19 Prevalence among Healthcare Workers. A Systematic Review and Meta-Analysis
Source: Int J Environ Res Public Health. 2021 Dec 23;19(1):146. doi: 10.3390/ijerph19010146 (PMC8750782; doi:10.3390/ijerph19010146)
Supplement: Supplementary file 1 [file ijerph-19-00146-s001.zip › Supplementary File S2.pdf]

Supplementary File S2: List of full text articles screened for eligibility.

1. Al Mahyijari N, Badahdah A, Khamis F. The psychological impacts of COVID-19: a study of frontline physicians and nurses in the Arab world. Irish Journal of Psychological Medicine. 2020:1-6.

2. Al Mutair A, Al Mutairi A, Ambani Z, Shamsan A, Almahmoud S, Alhumaid S. The impact of COVID-19 pandemic on the level of depression among health care workers: cross-sectional study. PeerJ. 2021;9:e11469.

3. Alajmi J, Jeremijenko AM, Abraham JC, Alishaq M, Concepcion EG, Butt AA, et al. COVID-19 infection among healthcare workers in a national healthcare system: The Qatar experience. International Journal of Infectious Diseases. 2020;100:386-9.

4. Algado-Sellés N, Gras-Valentí P, Chico-Sánchez P, Mora-Muriel JG, Soler-Molina VM, Hernández-Maldonado M, et al. Frequency, Associated Risk Factors, and Characteristics of COVID-19 Among Healthcare Personnel in a Spanish Health Department. American Journal of Preventive Medicine. 2020;59(6):e221-e9.

5. Ali S, Noreen S, Farooq I, Bugshan A, Vohra F. Risk Assessment of Healthcare Workers at the Frontline against COVID-19. Pakistan Journal of Medical Sciences. 2020;36(COVID19-S4).

6. Alshamrani MM, El-Saed A, Al Zunitan M, Almulhem R, Almohrij S. Risk of COVID-19 morbidity and mortality among healthcare workers working in a Large Tertiary Care Hospital. International Journal of Infectious Diseases. 2021;109:238-43.

7. Alshekaili M, Hassan W, Al Said N, Al Sulaimani F, Jayapal SK, Al-Mawali A, et al. Factors associated with mental health outcomes across healthcare settings in Oman during COVID-19: frontline versus non-frontline healthcare workers. BMJ Open. 2020;10(10):e042030.

8. Angamuthu N, Geraldine Gagasa E, Baker D, Tsui J, Evan D'Souza R. Transmission of infection among health care personnel performing surgical tracheostomies on COVID-19 patients. The Surgeon. 2021.

9. Arshad MS, Hussain I, Nafees M, Majeed A, Imran I, Saeed H, et al. <p>Assessing the Impact of COVID-19 on the Mental Health of Healthcare Workers in Three Metropolitan Cities of Pakistan</p>. Psychology Research and Behavior Management. 2020;Volume 13:1047-55.

10. Bai Y, Wang X, Huang Q, Wang H, Gurarie D, Ndeffo-Mbah M, et al. SARS-CoV-2 infection in health care workers: a retrospective analysis and model study. 2020.

11. Baker MA, Rhee C, Fiumara K, Bennett-Rizzo C, Tucker R, Williams SA, et al. COVID-19 infections among HCWs exposed to a patient with a delayed diagnosis of COVID-19. Infection Control & Hospital Epidemiology. 2020;41(9):1075-6.

12. Barrett ES, Horton DB, Roy J, Gennaro ML, Brooks A, Tischfield J, et al. Prevalence of SARS-CoV-2 infection in previously undiagnosed health care workers in New Jersey, at the onset of the U.S. COVID-19 pandemic. BMC Infectious Diseases. 2020;20(1).

13. Barry M, Robert AA, Temsah M-H, Abdul Bari S, Akhtar MY, Al Nahdi F, et al. COVID-19 Community Transmission among Healthcare Workers at a Tertiary Care Cardiac Center. Medical Sciences. 2021;9(3):49.

14. Basso T, Nordbø SA, Sundqvist E, Martinsen TC, Witsø E, Wik TS. Transmission of infection from non-isolated patients with COVID-19 to healthcare workers. Journal of Hospital Infection. 2020;106(4):639-42.

15. Baveja S, Karnik N, Natraj G, Natkar M, Bakshi A, Krishnan A. Rapid volunteer-based SARS-Cov-2 antibody screening among health care workers of a hospital in Mumbai, India. Indian Journal of Medical Sciences.72.

16. Baveja S, Karnik N, Natraj G, Natkar M, Bakshi A, Krishnan A. Rapid volunteer-based SARS-Cov-2 antibody screening among health care workers of a hospital in Mumbai, India. Indian Journal of Medical Sciences. 2020;72(3):148-54.

17. Bellizzi S, Fiamma M, Arru L, Farina G, Manca A. COVID-19: The daunting experience of healthcare workers in Sardinia, Italy. Infection Control & Hospital Epidemiology. 2020;41(9):1118-9.

18. Bi Q, Wu Y, Mei S, Ye C, Zou X, Zhang Z, et al. Epidemiology and transmission of COVID-19 in 391 cases and 1286 of their close contacts in Shenzhen, China: a retrospective cohort study. The Lancet Infectious Diseases. 2020;20(8):911-9.

19. Breazzano MP, Shen J, Abdelhakim AH, Glass LRD, Horowitz JD, Xie SX, et al. New York City COVID-19 resident physician exposure during exponential phase of pandemic. Journal of Clinical Investigation. 2020;130(9):4726-33.

20. Brown J, Gregson FKA, Shrimpton A, Cook TM, Bzdek BR, Reid JP, et al. A quantitative evaluation of aerosol generation during tracheal intubation and extubation. Anaesthesia. 2021;76(2):174-81.

21. Burrer SL, De Perio MA, Hughes MM, Kuhar DT, Luckhaupt SE, McDaniel CJ, et al. Characteristics of Health Care Personnel with COVID-19 — United States, February 12–April 9, 2020. MMWR Morbidity and Mortality Weekly Report. 2020;69(15):477-81.

22. Canova V, Lederer Schlpfer H, Piso RJ, Droll A, Fenner L, Hoffmann T, et al. Transmission risk of SARS-CoV-2 to healthcare workers –observational results of a primary care hospital contact tracing. Swiss Medical Weekly. 2020.

23. Chatterjee P, Anand T, Singh KJ, Rasaily R, Singh R, Das S, et al. Healthcare workers & SARS-CoV-2 infection in India: A case-control investigation in the time of COVID-19. The Indian journal of medical research. 2020;151(5):459-67.

24. Chen Y, Tong X, Wang J, Huang W, Yin S, Huang R, et al. High SARS-CoV-2 antibody prevalence among healthcare workers exposed to COVID-19 patients. Journal of Infection. 2020;81(3):420-6.

25. Cheng VC-C, Wong S-C, Yuen K-Y. Estimating Coronavirus Disease 2019 Infection Risk in Health Care Workers. JAMA Network Open. 2020;3(5):e209687.

26. Chow K, Aslam A, McClure T, Singh J, Burns J, McMillen T, et al. Risk of Healthcare-Associated Transmission of SARS-CoV-2 in Hospitalized Cancer Patients. Clinical Infectious Diseases. 2021.

27. Chowell G, Abdirizak F, Lee S, Lee J, Jung E, Nishiura H, et al. Transmission characteristics of MERS and SARS in the healthcare setting: a comparative study. BMC Medicine. 2015;13(1).

28. Contejean A, Leporrier J, Canouï E, Alby-Laurent F, Lafont E, Beaudeau L, et al. Comparing Dynamics and Determinants of Severe Acute Respiratory Syndrome Coronavirus 2 Transmissions Among Healthcare Workers of Adult and Pediatric Settings in Central Paris. Clinical Infectious Diseases. 2021;72(2):257-64.

29. Cummings DAT, Radonovich LJ, Gorse GJ, Gaydos CA, Bessesen MT, Brown AC, et al. Risk Factors for Healthcare Personnel Infection With Endemic Coronaviruses (HKU1, OC43, NL63, 229E): Results from the Respiratory Protection Effectiveness Clinical Trial (ResPECT). Clinical Infectious Diseases. 2020.

30. De Lusignan S, Dorward J, Correa A, Jones N, Akinyemi O, Amirthalingam G, et al. Risk factors for SARS-CoV-2 among patients in the Oxford Royal College of General Practitioners Research and Surveillance Centre primary care network: a cross-sectional study. The Lancet Infectious Diseases. 2020;20(9):1034-42.

31. Dev N, Meena RC, Gupta DK, Gupta N, Sankar J. Risk factors and frequency of COVID-19 among healthcare workers at a tertiary care centre in India: a case–control study. Transactions of The Royal Society of Tropical Medicine and Hygiene. 2021;115(5):551-6.

32. Dimcheff DE, Schildhouse RJ, Hausman MS, Vincent BM, Markovitz E, Chensue SW, et al. Seroprevalence of severe acute respiratory syndrome coronavirus-2 (SARS-CoV-2) infection among Veterans Affairs healthcare system employees suggests higher risk of infection when exposed to SARS-CoV-2 outside the work environment. Infection Control & Hospital Epidemiology. 2021;42(4):392-8.

33. Dutta U, Sachan A, Premkumar M, Gupta T, Sahoo S, Grover S, et al. Multidimensional dynamic healthcare personnel (HCP)-centric model from a low-income and middle-income country to support and protect COVID-19 warriors: a large prospective cohort study. BMJ Open. 2021;11(2):e043837.

34. El-Boghdadly K, Wong DJN, Owen R, Neuman MD, Pocock S, Carlisle JB, et al. Risks to healthcare workers following tracheal intubation of patients with COVID-19: a prospective international multicentre cohort study. Anaesthesia. 2020;75(11):1437-47.

35. Fell A, Beaudoin A, D'Heilly P, Mumm E, Cole C, Tourdot L, et al. SARS-CoV-2 Exposure and Infection Among Health Care Personnel — Minnesota, March 6–July 11, 2020. MMWR Morbidity and Mortality Weekly Report. 2020;69(43):1605-10.

36. Fowler RA, Guest CB, Lapinsky SE, Sibbald WJ, Louie M, Tang P, et al. Transmission of Severe Acute Respiratory Syndrome during Intubation and Mechanical Ventilation. American Journal of Respiratory and Critical Care Medicine. 2004;169(11):1198-202.

37. Fuereder T, Berghoff AS, Heller G, Haslacher H, Perkmann T, Strassl R, et al. SARS-CoV-2 seroprevalence in oncology healthcare professionals and patients with cancer at a tertiary care centre during the COVID-19 pandemic. ESMO Open. 2020;5(5):e000889.

38. Fukuda H, Seyama K, Ito K, Ai T, Nojiri S, Hori S, et al. SARS-CoV-2 seroprevalence in healthcare workers at a frontline hospital in Tokyo. Scientific Reports. 2021;11(1).

39. Fusco FM, Pisaturo M, Iodice V, Bellopede R, Tambaro O, Parrella G, et al. COVID-19 among healthcare workers in a specialist infectious diseases setting in Naples, Southern Italy: results of a cross-sectional surveillance study. Journal of Hospital Infection. 2020;105(4):596-600.

40. Garcia-Basteiro AL, Moncunill G, Tortajada M, Vidal M, Guinovart C, Jiménez A, et al. Seroprevalence of antibodies against SARS-CoV-2 among health care workers in a large Spanish reference hospital. Nature Communications. 2020;11(1).

41. Garduño-Orbe B, Sánchez-Rebolledo JM, Cortés-Rafael M, García-Jiménez Y, Perez-Ortiz M, Mendiola-Pastrana IR, et al. SARS-CoV-2 Reinfection among Healthcare Workers in Mexico: Case Report and Literature Review. Medicina. 2021;57(5):442.

42. Garzaro G, Clari M, Ciocan C, Grillo E, Mansour I, Godono A, et al. COVID-19 infection and diffusion among the healthcare workforce in a large university-hospital in northwest Italy. Med Lav. 2020;111(3):184-94.

43. Goenka M, Shah B, Goenka U, Das SS, Afzalpurkar S, Mukherjee M, et al. COVID -19 prevalence among health-care workers of Gastroenterology department: An audit from a tertiary-care hospital in India. JGH Open. 2021;5(1):56-63.

44. Gordon CL, Trubiano JA, Holmes NE, Chua KYL, Feldman J, Young G, et al. Staff to staff transmission as a driver of healthcare worker infections with COVID-19. Infection, Disease & Health. 2021.

45. Grant JJ, Wilmore SMS, McCann NS, Donnelly O, Lai RWL, Kinsella MJ, et al. Seroprevalence of SARS-CoV-2 antibodies in healthcare workers at a London NHS Trust. Infection Control & Hospital Epidemiology. 2021;42(2):212-4.

46. Guo X, Wang J, Hu D, Wu L, Gu L, Wang Y, et al. Survey of COVID-19 Disease Among Orthopaedic Surgeons in Wuhan, People's Republic of China. The Journal of bone and joint surgery American volume. 2020;102(10):847-54.

47. Hartmann S, Rubin Z, Sato H, O Yong K, Terashita D, Balter S. Coronavirus Disease 2019 (COVID-19) Infections Among Healthcare Workers, Los Angeles County, February–May 2020. Clinical Infectious Diseases. 2020.

48. Heinzerling A, Stuckey MJ, Scheuer T, Xu K, Perkins KM, Resseger H, et al. Transmission of COVID-19 to Health Care Personnel During Exposures to a Hospitalized Patient — Solano County, California, February 2020. MMWR Morbidity and Mortality Weekly Report. 2020;69(15):472-6.

49. Houlihan CF, Vora N, Byrne T, Lewer D, Kelly G, Heaney J, et al. Pandemic peak SARS-CoV-2 infection and seroconversion rates in London frontline health-care workers. The Lancet. 2020;396(10246):e6-e7.

50. Hughes MM, Groenewold MR, Lessem SE, Xu K, Ussery EN, Wiegand RE, et al. Update: Characteristics of Health Care Personnel with COVID-19 — United States, February 12–July 16, 2020. MMWR Morbidity and Mortality Weekly Report. 2020;69(38):1364-8.

51. Hunter E, Price DA, Murphy E, Van Der Loeff IS, Baker KF, Lendrem D, et al. First experience of COVID-19 screening of health-care workers in England. The Lancet. 2020;395(10234):e77-e8.

52. Hussen H, Aderaw Alemu Z. Risk of COVID-19 Infection and Associated Factors Among Healthcare Workers: A Cross-Sectional Study at Eka Kotebe Treatment Center in Ethiopia. International Journal of General Medicine. 2021;Volume 14:1763-72.

53. Iversen K, Bundgaard H, Hasselbalch RB, Kristensen JH, Nielsen PB, Pries-Heje M, et al. Risk of COVID-19 in health-care workers in Denmark: an observational cohort study. The Lancet Infectious Diseases. 2020;20(12):1401-8.

54. Jacob JT, Baker JM, Fridkin SK, Lopman BA, Steinberg JP, Christenson RH, et al. Risk Factors Associated With SARS-CoV-2 Seropositivity Among US Health Care Personnel. JAMA Network Open. 2021;4(3):e211283.

55. Kambhampati AK, O'Halloran AC, Whitaker M, Magill SS, Chea N, Chai SJ, et al. COVID-19–Associated Hospitalizations Among Health Care Personnel — COVID-NET, 13 States, March 1–May 31, 2020. MMWR Morbidity and Mortality Weekly Report. 2020;69(43):1576-83.

56. Kantele A, Lääveri T, Kareinen L, Pakkanen SH, Blomgren K, Mero S, et al. SARS-CoV-2 infections among healthcare workers at Helsinki University Hospital, Finland, spring 2020: Serosurvey, symptoms and risk factors. Travel Medicine and Infectious Disease. 2021;39:101949.

57. Karri K, Yarra P. Patient-facing healthcare workers and their families have a higher risk of hospital admission with COVID-19 than the general population. Evidence Based Nursing. 2021:ebnurs-2020-103.

58. Kassem AM, Talaat H, Shawky S, Fouad R, Amer K, Elnagdy T, et al. SARS-CoV-2 infection among healthcare workers of a gastroenterological service in a tertiary care facility. Arab Journal of Gastroenterology. 2020;21(3):151-5.

59. Keeley AJ, Evans C, Colton H, Ankcorn M, Cope A, State A, et al. Roll-out of SARS-CoV-2 testing for healthcare workers at a large NHS Foundation Trust in the United Kingdom, March 2020. Eurosurveillance. 2020;25(14).

60. Kim CJ, Choi WS, Jung Y, Kiem S, Seol HY, Woo HJ, et al. Surveillance of the Middle East respiratory syndrome (MERS) coronavirus (CoV) infection in healthcare workers after contact with confirmed MERS patients: incidence and risk factors of MERS-CoV seropositivity. Clinical Microbiology and Infection. 2016;22(10):880-6.

61. Kira IA, Shuwiekh HAM, Ashby JS, Elwakeel SA, Alhuwailah A, Sous MSF, et al. The Impact of COVID-19 Traumatic Stressors on Mental Health: Is COVID-19 a New Trauma Type. International Journal of Mental Health and Addiction. 2021.

62. Kishk RM, Nemr N, Aly HM, Soliman NH, Hagras AM, Ahmed AAA, et al. Assessment of potential risk factors for coronavirus disease-19 (COVID-19) among health care workers. Journal of infection and public health. 2021:S1876-0341(21)00191-X.

63. Kluytmans-Van Den Bergh MFQ, Buiting AGM, Pas SD, Bentvelsen RG, Van Den Bijllaardt W, Van Oudheusden AJG, et al. Prevalence and Clinical Presentation of Health Care Workers With Symptoms of Coronavirus Disease 2019 in 2 Dutch Hospitals During an Early Phase of the Pandemic. JAMA Network Open. 2020;3(5):e209673.

64. Ko J-H, Lee JY, Baek JY, Seok H, Park GE, Lee JY, et al. Serologic Evaluation of MERS Screening Strategy for Healthcare Personnel During a Hospital-Associated Outbreak. Infection Control & Hospital Epidemiology. 2017;38(2):234-8.

65. Korth J, Wilde B, Dolff S, Anastasiou OE, Krawczyk A, Jahn M, et al. SARS-CoV-2-specific antibody detection in healthcare workers in Germany with direct contact to COVID-19 patients. Journal of Clinical Virology. 2020;128:104437.

66. Kumar SS, Kumar A, Kirtana J, Singh AK, Shankar SH, Khan MA, et al. Risk factors and outcome among COVID-19 exposed and quarantined healthcare workers: A study on the status of existing practices of standard precautions. J Family Med Prim Care. 2020;9(10):5355-9.

67. Kursumovic E, Lennane S, Cook TM. Deaths in healthcare workers due to COVID-19: the need for robust data and analysis. Anaesthesia. 2020;75(8):989-92.

68. Lai J, Ma S, Wang Y, Cai Z, Hu J, Wei N, et al. Factors Associated With Mental Health Outcomes Among Health Care Workers Exposed to Coronavirus Disease 2019. JAMA Network Open. 2020;3(3):e203976.

69. Lai X, Wang M, Qin C, Tan L, Ran L, Chen D, et al. Coronavirus Disease 2019 (COVID-2019) Infection Among Health Care Workers and Implications for Prevention Measures in a Tertiary Hospital in Wuhan, China. JAMA Network Open. 2020;3(5):e209666.

70. Lan F-Y, Filler R, Mathew S, Buley J, Iliaki E, Bruno-Murtha LA, et al. COVID-19 symptoms predictive of healthcare workers' SARS-CoV-2 PCR results. PLOS ONE. 2020;15(6):e0235460.

71. Leeds C. COVID 19: Health care workers, risks, protection and transmission. The Lancet Regional Health - Europe. 2021;1:100022.

72. Leeds JS, Raviprakash V, Jacques T, Scanlon N, Cundall J, Leeds CM. Risk factors for detection of SARS-CoV-2 in healthcare workers during April 2020 in a UK hospital testing programme. EClinicalMedicine. 2020;26:100513.

73. Liu W, Tang F, Fang L-Q, De Vlas SJ, Ma H-J, Zhou J-P, et al. Risk factors for SARS infection among hospital healthcare workers in Beijing: a case control study. Tropical Medicine & International Health. 2009;14:52-9.

74. Loeb M, McGeer A, Henry B, Ofner M, Rose D, Hlywka T, et al. SARS among Critical Care Nurses, Toronto. Emerging Infectious Diseases. 2004;10(2):251-5.

75. Lynch JB, Davitkov P, Anderson DJ, Bhimraj A, Cheng VC-C, Guzman-Cottrill J, et al. Infectious Diseases Society of America Guidelines on Infection Prevention for Healthcare Personnel Caring for Patients With Suspected or Known Coronavirus Disease 2019. Clinical Infectious Diseases. 2020.

76. Ma H-j, Wang H-w, Fang L-q, Jiang J-f, Wei M-t, Liu W, et al. [A case-control study on the risk factors of severe acute respiratory syndromes among health care workers]. Zhonghua liu xing bing xue za zhi = Zhonghua liuxingbingxue zazhi. 2004;25(9):741-4.

77. Macintyre CR, Seale H, Yang P, Zhang Y, Shi W, Almatroudi A, et al. Quantifying the risk of respiratory infection in healthcare workers performing high-risk procedures. Epidemiology and Infection. 2014;142(9):1802-8.

78. Malik F-T-N, Ishraquzzaman M, Kalimuddin M, Choudhury S, Ahmed N, Badiuzzaman M, et al. Clinical Presentation, Management and In-Hospital Outcome of Healthcare Personnel With COVID-19 Disease. Cureus. 2020.

79. Maltezou HC, Dedoukou X, Tseroni M, Tsonou P, Raftopoulos V, Papadima K, et al. SARS-CoV-2 Infection in Healthcare Personnel With High-risk Occupational Exposure: Evaluation of 7-Day Exclusion From Work Policy. Clinical Infectious Diseases. 2020;71(12):3182-7.

80. Maltezou HC, Dedoukou X, Tsonou P, Tseroni M, Raftopoulos V, Pavli A, et al. Hospital factors associated with SARS-CoV-2 infection among healthcare personnel in Greece. Journal of Hospital Infection. 2021;109:40-3.

81. Manh Than H, Minh Nong V, Trung Nguyen C, Phu Dong K, Ngo HT, Thu Doan T, et al. <p>Mental Health and Health-Related Quality-of-Life Outcomes Among Frontline Health Workers During the Peak of COVID-19 Outbreak in Vietnam: A Cross-Sectional Study</p>. Risk Management and Healthcare Policy. 2020;Volume 13:2927-36.

82. Mansour M, Leven E, Muellers K, Stone K, Mendu DR, Wajnberg A. Prevalence of SARS-CoV-2 antibodies among healthcare workers at a tertiary academic hospital in New York City. Journal of general internal medicine. 2020;35:2485-6.

83. Martin C, Montesinos I, Dauby N, Gilles C, Dahma H, Van Den Wijngaert S, et al. Dynamics of SARS-CoV-2 RT-PCR positivity and seroprevalence among high-risk healthcare workers and hospital staff. Journal of Hospital Infection. 2020;106(1):102-6.

84. Mesnil M, Joubel K, Yavchitz A, Miklaszewski N, Devys J-M. Seroprevalence of SARS-Cov-2 in 646 professionals at the Rothschild Foundation Hospital (ProSeCoV study). Anaesthesia Critical Care & Pain Medicine. 2020;39(5):595-6.

85. Moscola J, Sembajwe G, Jarrett M, Farber B, Chang T, McGinn T, et al. Prevalence of SARS-CoV-2 Antibodies in Health Care Personnel in the New York City Area. JAMA. 2020;324(9):893.

86. Mutambudzi M, Niedzwiedz C, Macdonald EB, Leyland A, Mair F, Anderson J, et al. Occupation and risk of severe COVID-19: prospective cohort study of 120 075 UK Biobank participants. Occupational and Environmental Medicine. 2021;78(5):307-14.

87. Nguyen LH, Drew DA, Joshi AD, Guo C-G, Ma W, Mehta RS, et al. Risk of COVID-19 among frontline healthcare workers and the general community: a prospective cohort study. 2020.

88. Okediran JO, Ilesanmi OS, Fetuga AA, Onoh I, Afolabi AA, Ogunbode O, et al. The experiences of healthcare workers during the COVID-19 crisis in Lagos, Nigeria: A qualitative study. Germs. 2020;10(4):356-66.

89. Paderno A, Fior M, Berretti G, Schreiber A, Grammatica A, Mattavelli D, et al. SARS-CoV-2 Infection in Health Care Workers: Cross-sectional Analysis of an Otolaryngology Unit. Otolaryngology–Head and Neck Surgery. 2020;163(4):671-2.

90. Palladino R, Bollon J, Ragazzoni L, Barone-Adesi F. Excess Deaths and Hospital Admissions for COVID-19 Due to a Late Implementation of the Lockdown in Italy. International Journal of Environmental Research and Public Health. 2020;17(16):5644.

91. Parotto M, Cavallin F, Bryson GL, Chin KJ. Risks to healthcare workers following tracheal intubation of patients with known or suspected COVID-19 in Canada: data from the intubateCOVID registry. Canadian Journal of Anesthesia/Journal canadien d'anesthésie. 2021;68(3):425-7.

92. Pei L-y, Gao Z-c, Yang Z, Wei D-g, Wang S-x, Ji J-m, et al. Investigation of the influencing factors on severe acute respiratory syndrome among health care workers. Beijing da xue xue bao Yi xue ban = Journal of Peking University Health sciences. 2006;38(3):271-5.

93. Pessa Valente E, Cruz Vaz Da Costa Damásio L, Luz LS, Da Silva Pereira MF, Lazzerini M. COVID-19 among health workers in Brazil: The silent wave. Journal of Global Health. 2020;10(1).

94. Piccoli L, Ferrari P, Piumatti G, Jovic S, Rodriguez BF, Mele F, et al. Risk assessment and seroprevalence of SARS-CoV-2 infection in healthcare workers of COVID-19 and non-COVID-19 hospitals in Southern Switzerland. The Lancet Regional Health - Europe. 2021;1:100013.

95. Poletti P, Tirani M, Cereda D, Guzzetta G, Trentini F, Marziano V, et al. Seroprevalence of and Risk Factors Associated With SARS-CoV-2 Infection in Health Care Workers During the Early COVID-19 Pandemic in Italy. JAMA Network Open. 2021;4(7):e2115699.

96. Psichogiou M, Karabinis A, Pavlopoulou ID, Basoulis D, Petsios K, Roussos S, et al. Antibodies against SARS-CoV-2 among health care workers in a country with low burden of COVID-19. PLOS ONE. 2020;15(12):e0243025.

97. Raboud J, Shigayeva A, McGeer A, Bontovics E, Chapman M, Gravel D, et al. Risk Factors for SARS Transmission from Patients Requiring Intubation: A Multicentre Investigation in Toronto, Canada. PLoS ONE. 2010;5(5):e10717.

98. Ran L, Chen X, Wang Y, Wu W, Zhang L, Tan X. Risk Factors of Healthcare Workers With Coronavirus Disease 2019: A Retrospective Cohort Study in a Designated Hospital of Wuhan in China. Clinical Infectious Diseases. 2020;71(16):2218-21.

99. Rivett L, Sridhar S, Sparkes D, Routledge M, Jones NK, Forrest S, et al. Screening of healthcare workers for SARS-CoV-2 highlights the role of asymptomatic carriage in COVID-19 transmission. eLife. 2020;9.

100. Robles-Pérez E, González-Díaz B, Miranda-García M, Borja-Aburto VH. Infection and death by COVID-19 in a cohort of healthcare workers in Mexico. Scandinavian journal of work, environment & health. 2021;47(5):349-55.

101. Sampaio F, Sequeira C, Teixeira L. Impact of COVID-19 outbreak on nurses' mental health: A prospective cohort study. Environmental Research. 2021;194:110620.

102. Scales DC, Green K, Chan AK, Poutanen SM, Foster D, Nowak K, et al. Illness in Intensive Care Staff after Brief Exposure to Severe Acute Respiratory Syndrome. Emerging Infectious Diseases. 2003;9(10):1205-10.

103. Self WH, Tenforde MW, Stubblefield WB, Feldstein LR, Steingrub JS, Shapiro NI, et al. Seroprevalence of SARS-CoV-2 Among Frontline Health Care Personnel in a Multistate Hospital Network — 13 Academic Medical Centers, April–June 2020. MMWR Morbidity and Mortality Weekly Report. 2020;69(35):1221-6.

104. Shahid H, Haider MZ, Taqi M, Gulzar A, Zamani Z, Fatima T, et al. COVID-19 and Its Psychological Impacts on Healthcare Staff – A Multi-Centric Comparative Cross-Sectional Study. Cureus. 2020.

105. Sharma P, Chawla R, Bakshi R, Saxena S, Basu S, Bharti PK, et al. Seroprevalence of antibodies to SARS-CoV-2 and predictors of seropositivity among employees of a teaching hospital in New Delhi, India. Osong Public Health and Research Perspectives. 2021;12(2):88-95.

106. Sharma S, Mohindra R, Rana K, Suri V, Bhalla A, Biswal M, et al. Assessment of Potential Risk Factors for 2019-Novel Coronavirus (2019-nCov) Infection among Health Care Workers in a Tertiary Care Hospital, North India. Journal of Primary Care & Community Health. 2021;12:215013272110020.

107. Steensels D, Oris E, Coninx L, Nuyens D, Delforge M-L, Vermeersch P, et al. Hospital-Wide SARS-CoV-2 Antibody Screening in 3056 Staff in a Tertiary Center in Belgium. JAMA. 2020;324(2):195.

108. Stubblefield WB, Talbot HK, Feldstein LR, Tenforde MW, Ur Rasheed MA, Mills L, et al. Seroprevalence of SARS-CoV-2 Among Frontline Healthcare Personnel During the First Month of Caring for Patients With COVID-19—Nashville, Tennessee. Clinical Infectious Diseases. 2021;72(9):1645-8.

109. Sunil R, Bhatt MT, Bhumika TV, Thomas N, Puranik A, Chaudhuri S, et al. Weathering the Storm: Psychological Impact of COVID-19 Pandemic on Clinical and Nonclinical Healthcare Workers in India. Indian journal of critical care medicine : peer-reviewed, official publication of Indian Society of Critical Care Medicine. 2021;25(1):16-20.

110. Suzuki T, Hayakawa K, Ainai A, Iwata-Yoshikawa N, Sano K, Nagata N, et al. Effectiveness of personal protective equipment in preventing severe acute respiratory syndrome coronavirus 2 infection among healthcare workers. Journal of Infection and Chemotherapy. 2021;27(1):120-2.

111. Teleman MD, Boudville IC, Heng BH, Zhu D, Leo YS. Factors associated with transmission of severe acute respiratory syndrome among health-care workers in Singapore. Epidemiology and Infection. 2004;132(5):797-803.

112. Thibon P, Breton P, Mouet A, Bidon A, Haupais F, Darrigan C, et al. Healthcare associated coronavirus disease 2019 among health care workers in Normandy, France: a multi-center study. Infect Prev Pract. 2021;3(1):100109.

113. Treibel TA, Manisty C, Burton M, McKnight Á, Lambourne J, Augusto JB, et al. COVID-19: PCR screening of asymptomatic health-care workers at London hospital. The Lancet. 2020;395(10237):1608-10.

114. Trieu M-C, Bansal A, Madsen A, Zhou F, Sævik M, Vahokoski J, et al. SARS-CoV-2–Specific Neutralizing Antibody Responses in Norwegian Health Care Workers After the First Wave of COVID-19 Pandemic: A Prospective Cohort Study. The Journal of Infectious Diseases. 2021;223(4):589-99.

115. Villarreal J, Nieto SV, Vázquez F, Campo MTd, Mahillo I, de la Hoz RE. Time to a Negative SARS-CoV-2 PCR Predicts Delayed Return to Work After Medical Leave in COVID-19 Infected Health Care Workers. Journal of Occupational and Environmental Medicine. 2021.

116. Vimercati L, De Maria L, Quarato M, Caputi A, Stefanizzi P, Gesualdo L, et al. COVID-19 hospital outbreaks: Protecting healthcare workers to protect frail patients. An Italian observational cohort study. International Journal of Infectious Diseases. 2021;102:532-7.

117. Vlachoyiannopoulos P, Alexopoulos H, Apostolidi I, Bitzogli K, Barba C, Athanasopoulou E, et al. Anti-SARS-CoV-2 antibody detection in healthcare workers of two tertiary hospitals in Athens, Greece. Clinical Immunology. 2020;221:108619.

118. Wang G, Guan J-L, Zhu X-Q, Wang M-R, Fang D, Wen Y, et al. Infection, Screening, and Psychological Stress of Health-Care Workers With COVID-19 in a Nonfrontline Clinical Department. Disaster Medicine and Public Health Preparedness. 2020:1-7.

119. Wang X, Jiang X, Huang Q, Wang H, Gurarie D, Ndeffo-Mbah M, et al. Risk factors of SARS-CoV-2 infection in healthcare workers: a retrospective study of a nosocomial outbreak. Sleep Medicine: X. 2020;2:100028.

120. Wee LE, Sim JXY, Conceicao EP, Aung MK, Ng IM, Ling ML. Re: 'Personal protective equipment protecting healthcare workers in the Chinese epicenter of COVID-19' by Zhao et al. Clinical Microbiology and Infection. 2020;26(12):1719-21.

121. Wei X-S, Wang X-R, Zhang J-C, Yang W-B, Ma W-L, Yang B-H, et al. A cluster of health care workers with COVID-19 pneumonia caused by SARS-CoV-2. Journal of Microbiology, Immunology and Infection. 2021;54(1):54-60.

122. Wilder-Smith A, Teleman MD, Heng BH, Earnest A, Ling AE, Leo YS. Asymptomatic SARS Coronavirus Infection among Healthcare Workers, Singapore. Emerging Infectious Diseases. 2005;11(7):1142-5.

123. Wong T-W, Lee C-K, Tam W, Lau JT-F, Yu T-S, Lui S-F, et al. Cluster of SARS among Medical Students Exposed to Single Patient, Hong Kong. Emerging Infectious Diseases. 2004;10(2):269-76.

124. Yang Y, Lu L, Chen T, Ye S, Kelifa MO, Cao N, et al. Healthcare Worker's Mental Health and Their Associated Predictors During the Epidemic Peak of COVID-19. Psychology Research and Behavior Management. 2021;Volume 14:221-31.

125. Zhan M, Qin Y, Xue X, Zhu S. Death from Covid-19 of 23 Health Care Workers in China. New England Journal of Medicine. 2020;382(23):2267-8.

126. Zhang S, Guo M, Wu F, Xiong N, Ma Y, Wang Z, et al. Factors associated with asymptomatic infection in health-care workers with severe acute respiratory syndrome coronavirus 2 infection in Wuhan, China: a multicentre retrospective cohort study. Clinical Microbiology and Infection. 2020;26(12):1670-5.

127. Zhao Y, Liang W, Luo Y, Chen Y, Liang P, Zhong R, et al. Personal protective equipment protecting healthcare workers in the Chinese epicentre of COVID-19. Clinical Microbiology and Infection. 2020;26(12):1716-8.

128. Zheng C, Hafezi-Bakhtiari N, Cooper V, Davidson H, Habibi M, Riley P, et al. Characteristics and transmission dynamics of COVID-19 in healthcare workers at a London teaching hospital. Journal of Hospital Infection. 2020;106(2):325-9.
