# Supplementary material for: COVID-19 Prevalence among Healthcare Workers. A Systematic Review and Meta-Analysis
Source: Int J Environ Res Public Health. 2021 Dec 23;19(1):146. doi: 10.3390/ijerph19010146 (PMC8750782; doi:10.3390/ijerph19010146)
Supplement: Supplementary file 1 [file ijerph-19-00146-s001.zip › Supplementary File S5.pdf]

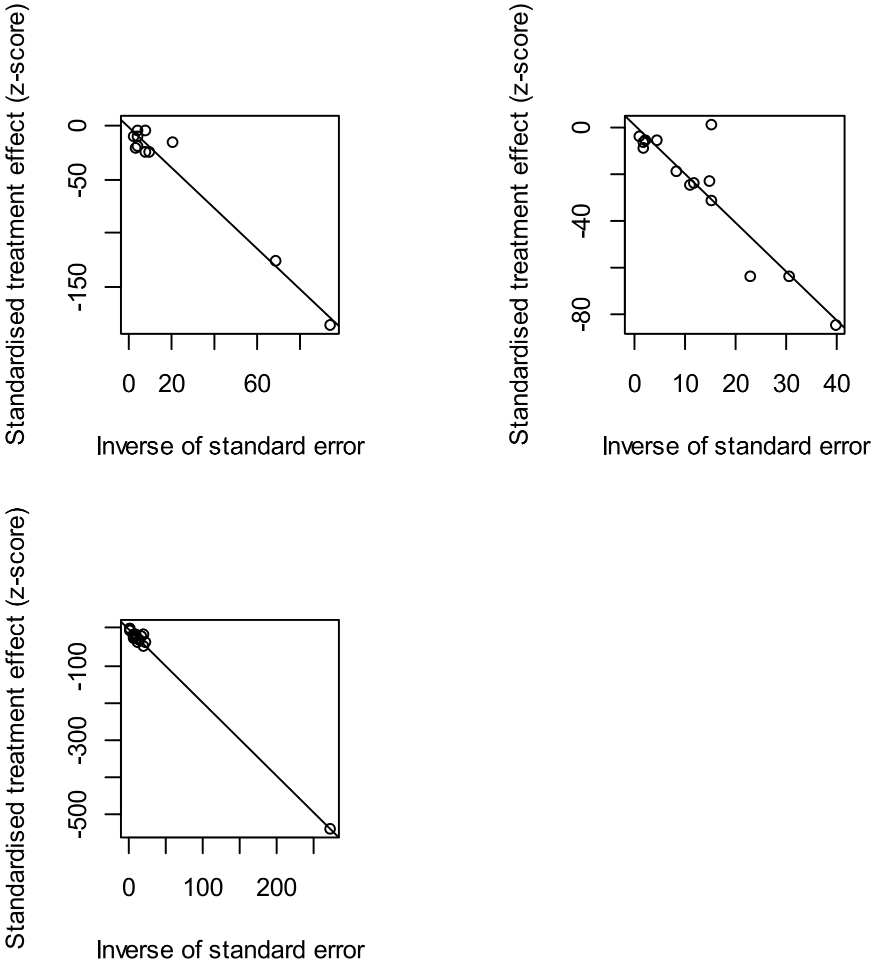

Supplementary File S5: Egger's plots for assessing the presence of publication bias for the meta-analysis of: 1). prevalence of COVID-19 among studies using the AB method of test *(left-top panel);* 2). prevalence of COVID-19 among studies using the PCR method of test *(right-top panel)*; and 3). Incidence rate of COVID-19 among studies using the PCR method of test *(left-bottom panel)*
