# Supplementary material for: COVID-19 Prevalence among Healthcare Workers. A Systematic Review and Meta-Analysis
Source: Int J Environ Res Public Health. 2021 Dec 23;19(1):146. doi: 10.3390/ijerph19010146 (PMC8750782; doi:10.3390/ijerph19010146)
Supplement: Supplementary file 1 [file ijerph-19-00146-s001.zip › Supplementary File S6.pdf]

Supplementary File S6: Presentation of findings for assessing and accounting for small-study effects

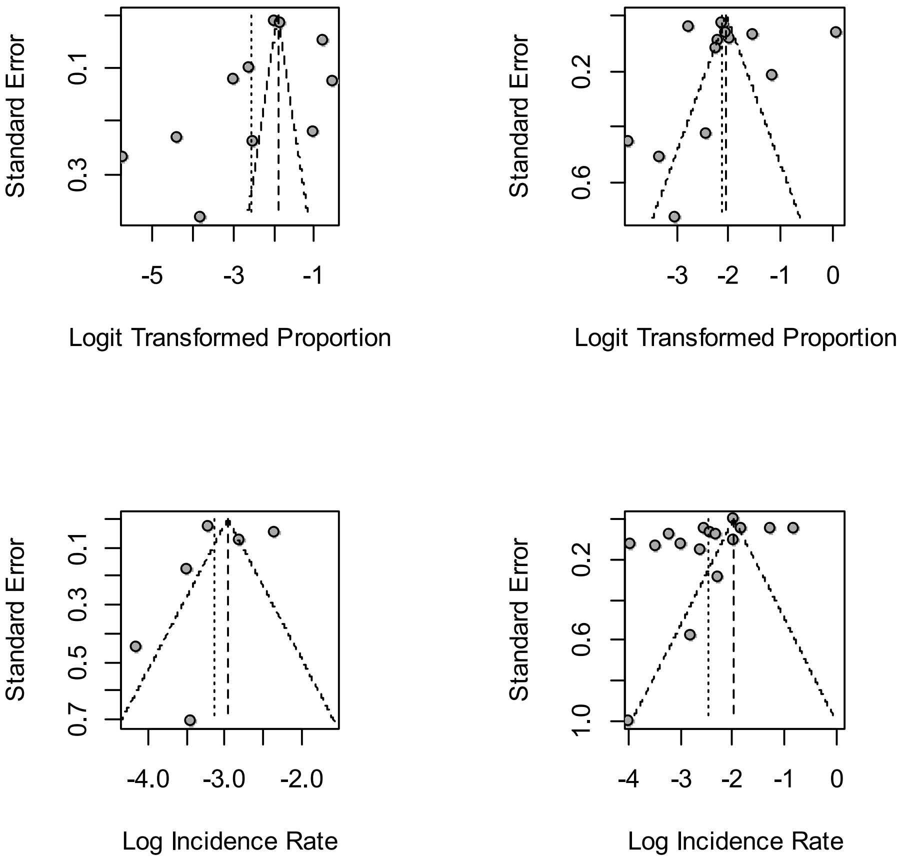

Figure S1. Funnel plots for assessing and accounting for small-study effects in the meta-analysis of: 1). prevalence of COVID-19 among studies using the AB method of test *(left-top panel);* 2). prevalence of COVID-19 among studies using the PCR method of test *(right-top panel)*; 3). Incidence rate of COVID-19 among studies using the AB method of test *(left-bottom panel);* 2). Incidence rate of COVID-19 among studies using the PCR method of test; *(right-bottom panel).*

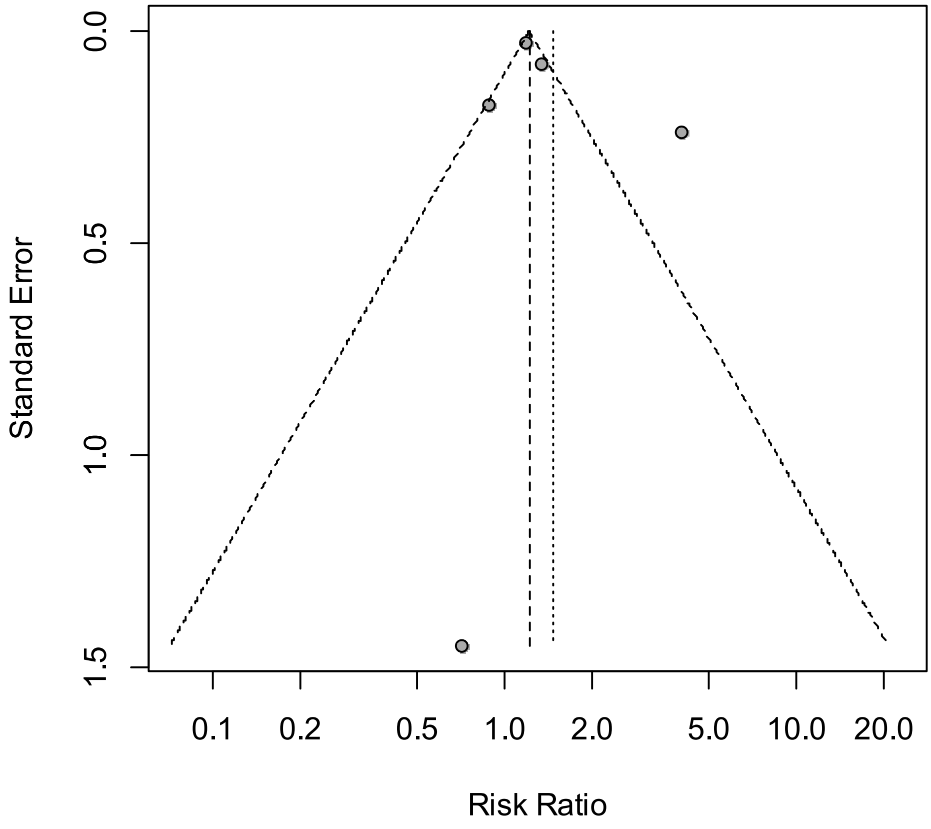

Figure S2. Funnel plots for assessing and accounting for small-study effects in the meta-analysis of risk of COVID-19 between patient and non-patient facing HCWs.
