# Supplementary figures and images for: COVID-19 Prevalence among Healthcare Workers. A Systematic Review and Meta-Analysis
Source: Int J Environ Res Public Health. 2021 Dec 23;19(1):146. doi: 10.3390/ijerph19010146 (PMC8750782; doi:10.3390/ijerph19010146)

# Supplementary File S1. PRISMA flow diagram

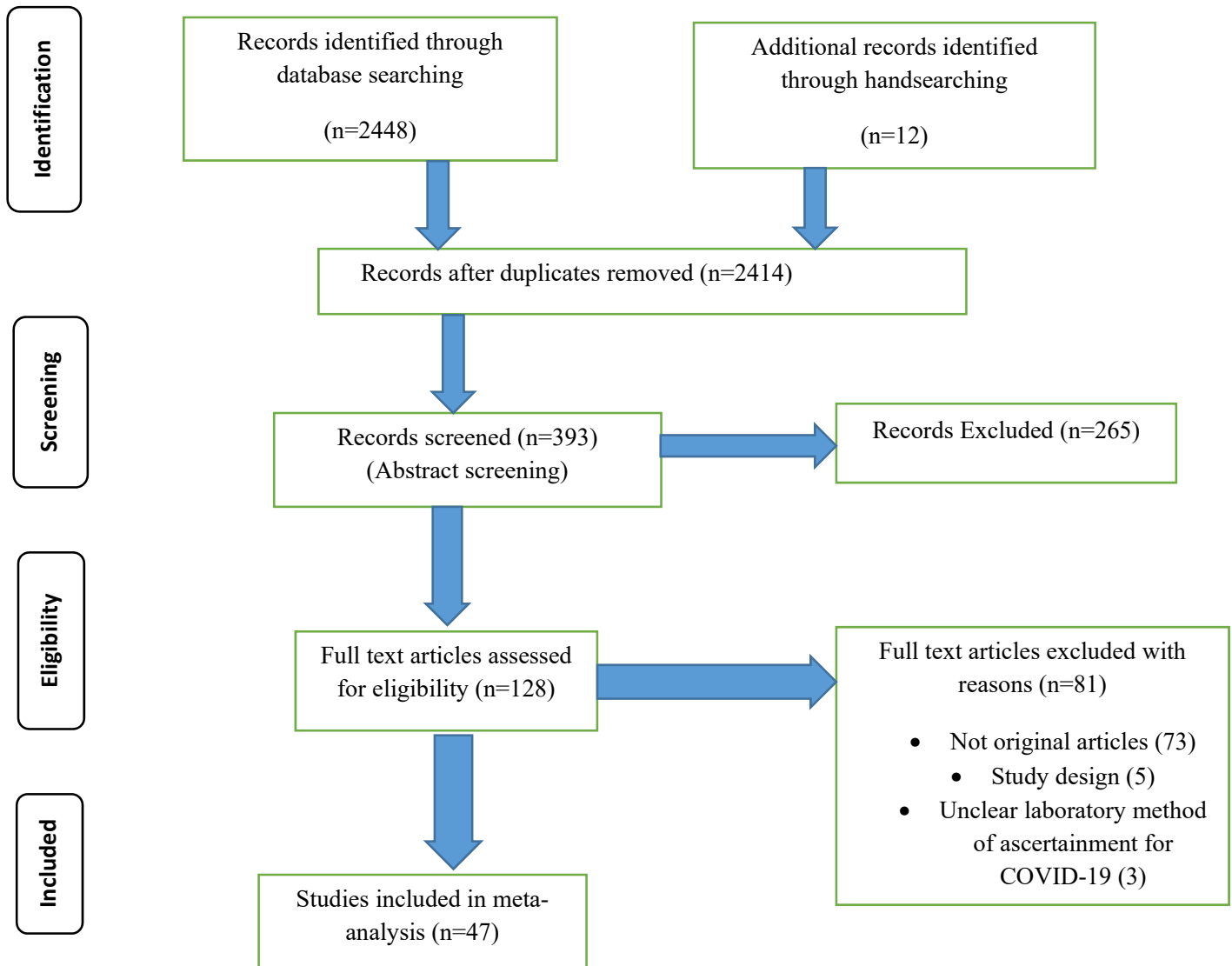

Supplement: Supplementary file 1 [file ijerph-19-00146-s001.zip › Supplementary File S1.pdf]
